# Functional Genomics and Phylogenetic Evidence Suggest Genus-Wide Cobalamin Production by the Globally Distributed Marine Nitrogen Fixer *Trichodesmium*

**DOI:** 10.3389/fmicb.2018.00189

**Published:** 2018-02-13

**Authors:** Nathan G. Walworth, Michael D. Lee, Christopher Suffridge, Pingping Qu, Fei-Xue Fu, Mak A. Saito, Eric A. Webb, Sergio A. Sañudo-Wilhelmy, David A. Hutchins

**Affiliations:** ^1^Department of Biological Sciences, University of Southern California, Los Angeles, CA, United States; ^2^Marine Chemistry and Geochemistry Department, Woods Hole Oceanographic Institution, Woods Hole, MA, United States

**Keywords:** cobalamin, trichodesmium, Vitamin B_12_, iron limitation, Cyanobacteria, BluB gene, nitrogen fixation

## Abstract

Only select prokaryotes can biosynthesize vitamin B_12_ (i.e., cobalamins), but these organic co-enzymes are required by all microbial life and can be vanishingly scarce across extensive ocean biomes. Although global ocean genome data suggest cyanobacteria to be a major euphotic source of cobalamins, recent studies have highlighted that >95% of cyanobacteria can only produce a cobalamin analog, pseudo-B_12_, due to the absence of the BluB protein that synthesizes the α ligand 5,6-dimethylbenzimidizole (DMB) required to biosynthesize cobalamins. Pseudo-B_12_ is substantially less bioavailable to eukaryotic algae, as only certain taxa can intracellularly remodel it to one of the cobalamins. Here we present phylogenetic, metagenomic, transcriptomic, proteomic, and chemical analyses providing multiple lines of evidence that the nitrogen-fixing cyanobacterium *Trichodesmium* transcribes and translates the biosynthetic, cobalamin-requiring BluB enzyme. Phylogenetic evidence suggests that the *Trichodesmium* DMB biosynthesis gene, *bluB*, is of ancient origin, which could have aided in its ecological differentiation from other nitrogen-fixing cyanobacteria. Additionally, orthologue analyses reveal two genes encoding iron-dependent B_12_ biosynthetic enzymes (cbiX and isiB), suggesting that iron availability may be linked not only to new nitrogen supplies from nitrogen fixation, but also to B_12_ inputs by *Trichodesmium*. These analyses suggest that *Trichodesmium* contains the genus-wide genomic potential for a previously unrecognized role as a source of cobalamins, which may prove to considerably impact marine biogeochemical cycles.

## Introduction

Marine cyanobacteria and eukaryotic algae are estimated to be responsible for up to 50% of global carbon fixation, and can be limited by both macronutrients (e.g., nitrogen and phosphorus) and micronutrients (e.g., iron) (Field et al., 1998; Arrigo, 2005; Hutchins et al., 2009; Hutchins and Boyd, 2016). Organic coenzymes known as B-vitamins have also been implicated to limit primary production and influence microbial community structure (Panzeca et al., 2006; Sañudo-Wilhelmy et al., 2014; Suffridge et al., 2017). B-vitamins are soluble, non-protein molecules that bind to enzymes to increase reaction rates and are required for essential cellular processes such as DNA repair, redox reactions, photosynthesis, and carbon fixation (Monteverde et al., 2016). Most eukaryotic algae (Droop, 2007) and many heterotrophic bacteria (Giovannoni et al., 2005) have obligate B-vitamin requirements (e.g., auxotrophy) for processes like reductive dehalogenation and fatty acid biosynthesis, with growth requiring assimilation via exogenous sources including breakdown of vitamin-containing cells and/or interactions with vitamin-producing bacteria and archaea (Stadtman et al., 1960; Mohn and Tiedje, 1992; Bertrand et al., 2015; Heal et al., 2016). Microbes that do not have an absolute requirement for B_12_ employ B_12_-independent versions of certain enzymes such as the B_12_-independent methionine synthase (MetE) and the B_12_-independent radical SAM DNA methyltransferases (Heal et al., 2016).

**Figure 1:**
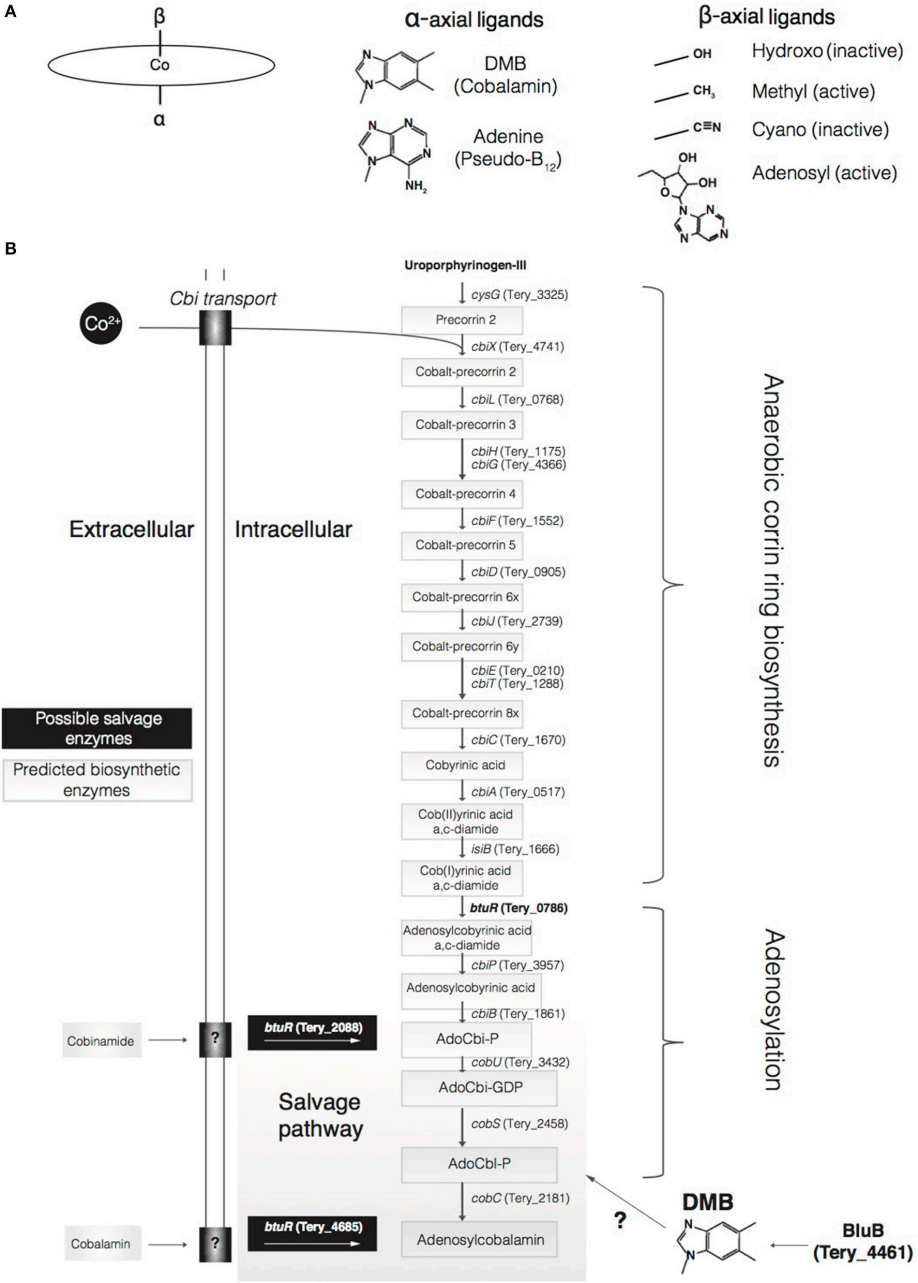
Diagram of different forms of cobalamin, Psuedo-B_12_ and proposed cobalamin biosynthetic and salvage pathways. **(A)** The ellipse represents the cobalt-containing corrin ring, and listed are the various α-axial and β-axial ligands. **(B)** Displayed is the fully predicted pathway for B_12_ biosynthesis including potential salvage pathways.

Some chemical form of vitamin B_12_ is required by all microbial life for a range of functions, including methionine biosynthesis, ribonucleotide reduction, photoregulation, and various one-carbon metabolisms (Sañudo-Wilhelmy et al., 2014; Fang et al., 2017). Vitamin B_12_ is a general term referring to cobalt-containing corrinoids (i.e., molecules containing a corrin ring) that contain upper (β) and lower (α) axial ligands to the cobalt ion that can vary depending on functionality (Eschenmoser and Wintner, 1977; Warren et al., 2002; Helliwell et al., 2016). A primary type of B_12_, cobalamin (CBL), is a complex coenzyme with an α ligand of 5,6-dimethylbenzimidizole (DMB), and a β ligand of either an adenosyl-, methyl-, cyanide-, or hydroxyl-group (Ado-, Me-, CN-, or OH-; Heal et al., 2016; Figure 1). For example, methylcobalamin (Me-CBL) has a methyl group as its β ligand and is involved in methylation reactions, whereas adenosylcobalamin (Ado-CBL, coenzyme B_12_) has an adenosyl group (5–deoxyadenosine) and is involved in radical-based rearrangements and reductions (Banerjee and Ragsdale, 2003).

Many marine microbes have an absolute requirement for B_12_ (Bertrand et al., 2013), and locally produced cobalamin is likely the predominant source for microbial assimilation due to its short (hours to days) residence time in the surface ocean (Carlucci et al., 1969; Bertrand et al., 2015). Those microbes that can grow without cobalamins must do so at a metabolic cost, using an alternative methionine synthase (MetE) that is relatively less-efficient than the B_12_-dependent MetH enzyme (Helliwell et al., 2016). These biochemical constraints and environmental interactions highlight B_12_ as a potential driver of ecological niche partitioning in marine environments. Thaumarchaeota (Doxey et al., 2015; Santoro et al., 2015) and both heterotrophic bacteria (Croft et al., 2005) and cyanobacteria (Bonnet et al., 2010) are hypothesized to be major cobalamin producers in the ocean, thereby potentially modulating overall primary productivity. However, recent studies have indicated that most cyanobacteria produce a cobalamin analog, pseudo-B_12_ (Taga and Walker, 2008), where adenine substitutes for DMB as the α ligand (Heal et al., 2016; Helliwell et al., 2016) (Figure 1). The compound DMB is synthesized aerobically by the BluB enzyme, or anaerobically via enzymes of the *bza* operon (Hazra et al., 2015; Mehta et al., 2015). A recent assessment found that only one cyanobacterial genome out of 255 contained an annotated *bluB* gene (Heal et al., 2016), and a targeted search of 118 cyanobacterial genomes found it in only five species, while none contain the *bza* operon (Helliwell et al., 2016). Indeed, numerous BluB-lacking strains of the globally distributed *Synechococcus* and *Prochlorococcus* genera solely biosynthesize pseudo-B_12_ (Heal et al., 2016; Helliwell et al., 2016). Additionally, pseudo-B_12_ is used less efficiently than cobalamin for several B_12_-dependent algae, and only certain algae can intracellularly remodel (i.e., salvage) pseudo-B_12_ to cobalamin in the presence of exogenous DMB (Helliwell et al., 2016). Cultured algae whose growth could be rescued with supplemented pseudo-B_12_ + DMB could not grow in natural seawater supplemented with pseudo-B_12_ alone, thereby indicating DMB concentrations to be insufficient in certain habitats (Helliwell et al., 2016). In contrast, *bluB*-containing bacteria and archaea synthesize only cobalamin (Heal et al., 2016). Although BluB synthesizes DMB, the enzyme CobT has been found to be necessary for its activation across most microbial phyla studied to date (Croft et al., 2006; Escalante-Semerena, 2007; Yan et al., 2018). The bluB-containing Thaumarchaeota however solely produce cobalamin despite lacking cobT, demonstrating that DMB activation may occur through a similar enzyme with high sequence divergence or through a different, yet unknown, genetic mechanism (Heal et al., 2016). *Trichodesmium* may also employ a different activation mechanism than the previously identified CobT, as described below.

Heal et al. (2016) detected both particulate pseudo-B_12_ and cobalamin in the North Pacific surface ocean at similar concentrations (0.01 to 0.1 pM L^−1^), yet solely cobalamin beneath the photic zone. This co-occurrence at the surface suggests that *bluB*-lacking cyanobacteria synthesize pseudo-B_12_ in the presence of cobalamin, and that cobalamin in the surface ocean could be a result of either *de novo* synthesis by heterotrophic microbes, or remodeled pseudo-B_12_ originally from cyanobacteria. Hence, cyanobacteria may gain a competitive advantage by producing and solely requiring pseudo-B_12_ for growth, as this avoids them directly supplying other cobalamin-requiring photoautotrophs.

In contrast, marine nitrogen-fixing cyanobacteria (diazotrophs) directly supply both fixed carbon and nitrogen, thereby impacting marine primary productivity and biogeochemical cycling (Falkowski et al., 1998; Sohm et al., 2011). The globally distributed colony-forming diazotroph, *Trichodesmium*, is among the most important global contributors of bioavailable nitrogen in the oligotrophic oceans with some estimates suggesting it to make up as much as half of total N_2_ fixation in the vast subtropical gyre regions (Zehr, 2011; Hutchins et al., 2015). *Trichodesmium* can form extensive, recurring blooms in the Arabian and Red seas and Atlantic and Pacific oceans (Walworth et al., 2015). It serves as a millimeter-sized substrate for prokaryotic and eukaryotic microbial consortia (Bergman et al., 2013; Lee et al., 2017a) and also provides organic metabolites via excretion (Capone et al., 1994). Another common albeit less prevalent marine diazotroph, the unicellular genus *Crocosphaera*, excretes B-vitamins in culture at rates that exceed those of non-diazotrophic cyanobacteria (Bonnet et al., 2010), and recent studies have shown excretion of B-vitamin precursors in marine heterotrophic bacteria known to associate with phytoplankton blooms (Wienhausen et al., 2017). Despite intensive study on the global distribution and ecophysiology of *Trichodesmium*, knowledge about its capacity to biosynthesize and salvage critical cobalamin-type coenzymes is lacking.

Recently, we conducted a 7-year experimental evolution study adapting *Trichodesmium erythraeum* IMS101 (IMS101) to high CO_2_ under multiple limiting nutrient regimes. We generated a series of functional genomic datasets to explore both its molecular evolution (Hutchins et al., 2015; Walworth et al., 2016) and its ecological relationship with its epibiotic consortia (Lee et al., 2017a). While not originally intended to examine B-vitamin metabolism, we conducted a meta-analysis of our functional genomic datasets combined with other *Trichodesmium* metagenomic (Walworth et al., 2015), and phylogenetic data, which revealed strong evidence that the *Trichodesmium* genus can biosynthesize and salvage cobalamin *in situ*. Additionally, we substantiate molecular results by measuring intracellular cobalamin concentrations from our remaining long-term *Trichodesmium* samples using liquid chromatography coupled to mass spectrometry (LCMS) (Suffridge et al., 2017), thereby providing evidence of metabolized cobalamin. Corrin ring biosynthesis and adenosylation is conserved among cyanobacteria (Heal et al., 2016) indicating their presence within the phylum when it originally diverged. We conducted further phylogenetic analyses to explore the potential alternative origins of the *bluB* gene necessary for DMB biosynthesis, which may help elucidate B_12_ niche partitioning of *Trichodesmium* from co-occurring, sympatric cyanobacterial taxa including other diazotrophs and the cosmopolitan non-N_2_-fixing genera, *Prochlorococcus* and *Synechococcus*. We also describe B_12_-associated transcripts deriving from the *Trichodesmium* bacterial epibiotic community to examine a possible role of intra-colony cobalamin cycling. This previously unreported (Capone et al., 1997; Sohm et al., 2011; Bergman et al., 2013; Walworth et al., 2015) potential source of cobalamin deriving from either *Trichodesmium*, its epibionts, or both provide evidence for yet another critical role that *Trichodesmium* may serve by supplying a potentially limiting organic micronutrient to the surrounding microbial community. Further, comparative phylogenetic analyses highlight divergent sequence domains of cyanobacterial B_12_ biosynthetic and salvage pathways relative to other sequenced prokaryotes. Finally, we use comparative orthologue analyses to highlight the possible association of two B_12_ biosynthetic genes with environmental iron supply, suggesting simultaneous influence of both *Trichodesmium* nitrogen fixation and B_12_ production by this limiting trace element.

## Materials and methods

### Sequence analyses

Gene sequences for the cultured *T. erythraeum* isolates IMS101 and 2175 were downloaded from the Integrated Microbial Genomes (IMG) website (https://img.jgi.doe.gov/), and *Trichodesmium* environmental metagenomic sequence data was used from Walworth et al. 2015 (Walworth et al., 2015). Sequences were searched against the RefSeq protein database (Tatusova et al., 2015) using the BLASTP algorithm (Altschul et al., 1990), and all high-scoring pairs were retained if the aligned portion spanned >70% of the original query length with an evalue < 10^−5^. Duplicate sequences were removed with USEARCH (Edgar, 2010). All sequences were aligned with MUSCLE v3.8.31 with default settings (Edgar, 2004), and spurious sequences and poorly aligned regions were removed with trimAl 1.2rev59 (Capella-Gutiérrez et al., 2009). RAxML (Stamatakis, 2015) was used for all maximum likelihood phylogenetic analyses with the following settings: -f a -p 12345 -m PROTCATLG -N 100 -x 12345.

### Culturing and molecular analyses

Culturing and sampling for RNA and protein of *T. erythraeum* IMS101 was done as previously described (Walworth et al., 2015). Briefly, semi-continuous cultures growing in replete Aquil media (0.37 nM of added B_12_ as cyanocobalamin) without added fixed nitrogen and a 12:12 light:dark cycle (light intensity of 120 μmol photons per meter squared per second) at 26°C were filtered in biological duplicate at midday onto 5 um polycarbonate filters (Whatman), immediately flash frozen, and stored in liquid nitrogen. In an additional set of experiments, triplicate cultures were grown for ~20 generations using the same growth medium, but without added cyanocobalamin, to determine if IMS101 can grow in culture without a supplementary source of B_12._

RNA was extracted using the Ambion MirVana miRNA Isolation Kit (Thermo Fisher Scientific) in an RNAse free environment as per the manufacturer's instructions, followed by Ambion's Turbo DNA-free kit to degrade trace amounts of DNA. RNA was then submitted to the UC San Diego Institute for Genomic Medicine (IGM) core for library preparation and sequencing (http://igm.ucsd.edu/genomics/services.shtml). Briefly, rRNA removal and library construction was done with the TruSeq Stranded RNA Library Prep kit (Illumina), and multiplexed libraries were sequenced using the Illumina Hi-Seq yielding single-end, 50-base pair reads. Raw fastq files were quality trimmed and filtered with Trimmomatic version 0.35 (Bolger et al., 2014) with the following settings: SE -threads 35 -phred33 LEADING:3 TRAILING:3 SLIDINGWINDOW:4:15 MINLEN:35. Trimmed fastq files were then mapped onto IMS101 IMG-called genes (https://img.jgi.doe.gov/) using Bowtie2 v2.2.5 with default settings (Langmead and Salzberg, 2012), and the resulting count matrix was subjected to TMM normalization using edgeR (Robinson et al., 2010). Please see Supplementary Information for consortia read processing and annotation. Protein spectral counts were downloaded directly from a previously published proteome study using these same IMS101 cell lines (Walworth et al., 2016).

### Particulate B-vitamin analyses

Cobalamins were extracted from biological duplicate frozen filters from long-term IMS101 cultures grown in seawater medium containing containing 0.37 nM cyanocobalamin (Walworth et al., 2016), as previously described (Suffridge et al., 2017). See Supplementary Information for a brief method description.

## Results and discussion

### Evidence for cobalamin biosynthesis in *Trichodesmium*

**Figure 2:**
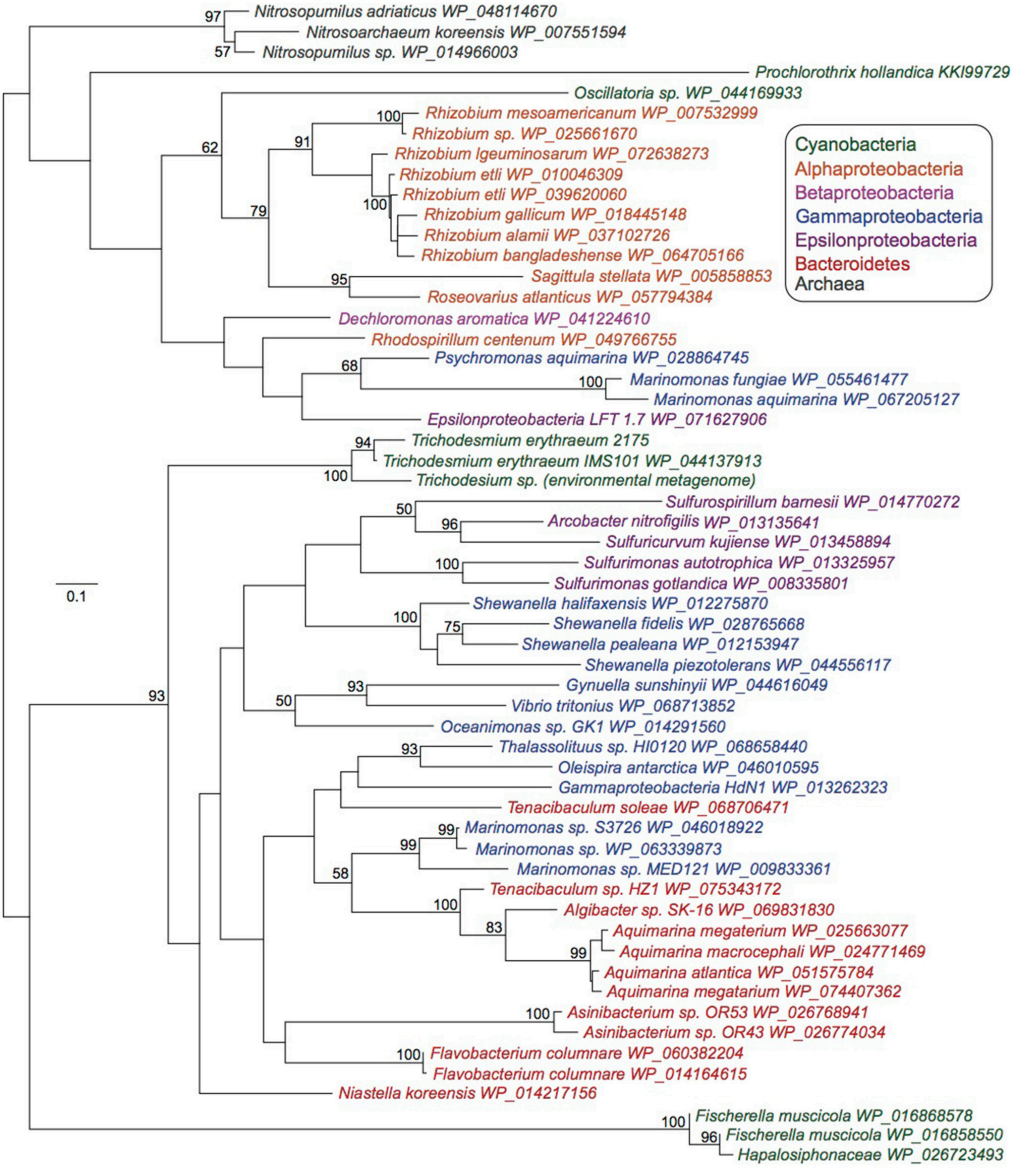
Maximum likelihood phylogenetic tree of *bluB* homologs. Unrooted, 100 bootstraps, values > 50 noted. Scale bar represents average substitutions per site.

The *bluB* gene required for aerobic DMB synthesis for cobalamin production has only been detected in five cyanobacterial species (Helliwell et al., 2016), none of which are abundant or quantitatively important in marine ecosystems. Phylogenetic analysis reveals that *Trichodesmium bluB* homologs from two *T. erythraeum* strains isolated 10 years apart (IMS101 and 21-75) (Walworth et al., 2015) and an environmental *Trichodesmium* metagenomic sample form their own clade among heterotrophic bacteria (Figure 2). While it is difficult to speculate as to whether the other cyanobacteria possessing *bluB* homologs acquired them independently or if other cyanobacteria have selectively lost their copies, this analysis suggests the possibility that *bluB* has been frequently horizontally transferred and retained between many members of the Proteobacteria and Bacteroidetes phylum (Figure 2).

**Figure 3:**
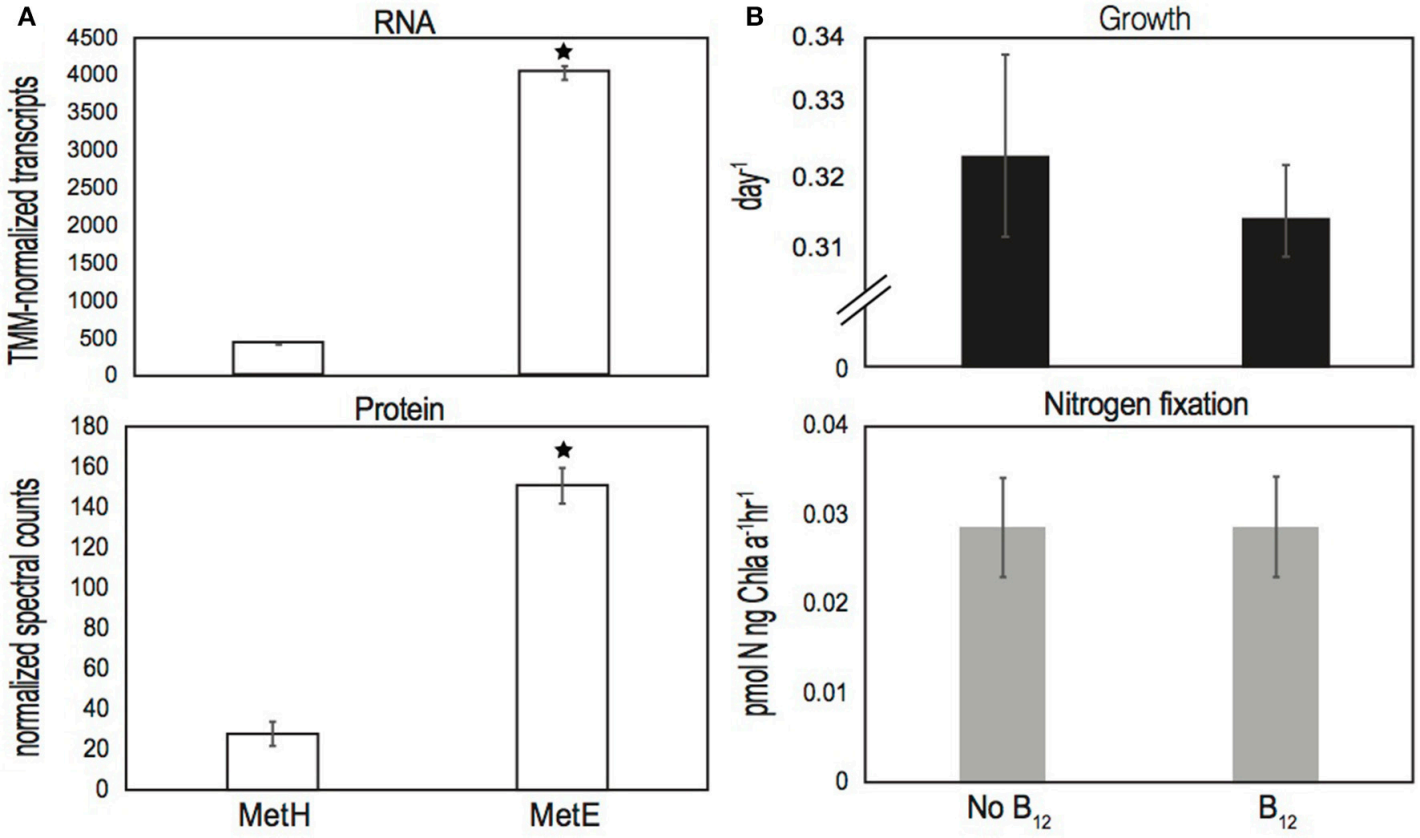
**(A)** Normalized RNA transcript and protein spectral counts of MetH and MetE from cultures grown in the presence of cyanocobalamin, respectively, and **(B)** growth and N_2_ fixation data of IMS101 after ~20 generations of growth either with and without added cyanocobalamin. Error bars represent standard deviations of three biological replicates per treatment, and an asterisk (^*^) denotes significant differences (*p* < 0.05). Units of N_2_ fixation are in picomole nitrogen per nanogram of chlorophyll-a per hour.

RNA and protein extracted midday from long-term IMS101 cultures growing semi-continuously (Walworth et al., 2016) demonstrate *bluB* to be both actively transcribed and translated into protein (Table S1). The detection of the BluB protein provides strong evidence for active DMB biosynthesis because although genes can be constitutively transcribed at basal levels, bioenergetic investment dramatically increases at the translational level (Lynch and Marinov, 2015). Additionally, since half-lives of bacterial mRNA transcripts are typically short-lived relative to proteins (Rauhut and Klug, 1999), detection of both *bluB* transcripts and BluB proteins lend further evidence of persistent activity. Furthermore, all B_12_ biosynthetic genes were actively expressed, of which numerous corresponding protein products were also detected (Table S1). Interestingly, the B_12_-dependent (*metH*) and B_12_-independent (*metE*) methionine synthases were also both transcribed and translated in the presence of exogenously supplied cobalamin with *metE* transcripts and protein levels being ~10X (*p* < 10^−4^) and ~5X (*p* < 0.05) higher, respectively, than those of *metH* (Figure 3A). Prior analysis of bacterial MetE suggests reduced enzyme efficiency relative to MetH (Bertrand et al., 2013; Helliwell et al., 2016), which may explain the increased abundances of MetE transcripts and protein relative to those of MetH. Nonetheless, it is noteworthy that transcripts and proteins of both forms were detected in the presence of cobalamin, suggesting *Trichodesmium* to persistently utilize both versions of the enzyme irrespective of environmental cobalamin supply.

Additionally, we applied a recently developed method that measures particulate B_12_ concentrations directly from cell lysates to the remaining samples from the long-term *Trichodesmium* experiment (Walworth et al., 2016), which detected intracellular cobalamins in IMS101 trichomes at a concentration of ~58 pM (Figure S1) (Suffridge et al., 2017). Hence, these measurements suggest the ability of IMS101 to metabolize cobalamins either from salvage or biosynthesis pathways. To assess if physiology is impacted without exogenously supplied B_12_, we performed additional physiology experiments examining growth and nitrogen fixation rates of IMS101 with and without added cobalamin (Methods) and found no difference in either condition (Figure 3B; *p* > 0.05). Hence, *Trichodesmium* does not require added B_12_ in culture medium, which further supports an ability to biosynthesize and/or salvage B_12_.

Notably, both *Trichodesmium* trichomes and colonies (i.e., aggregated trichomes) harbor physically attached microbial consortia comprised of hetero- and phototrophs, including other cyanobacteria, both in culture (Lee et al., 2017a,b) and *in situ* (Hewson et al., 2009; Rouco et al., 2016). To our knowledge no axenic *Trichodesmium* cultures currently exist. Because it is therefore impossible to absolutely differentiate potential sources of cobalamin deriving from either IMS101 itself or its microbial epibionts, the active production of both the BluB protein and its transcripts in *Trichodesmium* cells provides the clearest evidence for cobalamin production in IMS101. Additionally, although the biomass and proportional expression of other cyanobacteria is exceedingly low relative to that of *Trichodesmium* in colonies (Lee et al., 2017a; 75–80% of RNA sequencing reads mapped to the IMS101 genome with the remaining mapping to the rest of the consortia), it would also be prohibitively difficult to completely disentangle sources of pseudo-B_12_ in the particulate fraction if other cyanobacteria were indeed attached to IMS101.

As in naturally occurring colonies isolated *in situ* (Hewson et al., 2009), our IMS101 cultures contain trace amounts of *Synechococcus* (Lee et al., 2017a), thereby obstructing the ability to specifically detect pseudo-B_12_ from *Trichodesmium*. We also detected transcripts of B_12_ biosynthesis/salvage genes deriving from both *Synechococcus* and heterotrophic genera in the *Trichodesmium* epibiotic community (Table S1). Interestingly, only the *btuR* gene was detected in heterotrophic bacterial transcripts, which can be involved in either biosynthesis or salvage pathways (see below) while both *btuR* and the B_12_ biosynthetic gene, *cbiB*, was detected in cyanobacterial transcripts suggesting production of pseudo-B_12_ by *Synechococcus*. However, since no other B_12_ biosynthesis genes were detected in heterotrophic transcripts other than *btuR*, this opens the door for further investigation pertaining to the sources and sinks of cobalamins between *Trichodesmium* and its heterotrophic epibionts. A recent genome analysis of IMS101 heterotrophic epibionts revealed that an associated *Alteromonas macleodii* genome from this isolate is indeed a B_12_ auxotroph (Lee et al., 2017a). Moreover, the detection of consortia *btuR* genes may have implications for the use of organically complexed cobalt, which dominates cobalt speciation in most euphotic zone environments (Saito et al., 2005). Additional studies with *Trichodesmium* colonies devoid of other cyanobacteria coupled to direct measurements of both cobalamin and pseudo-B_12_ are necessary to determine whether these two forms are simultaneously produced by IMS101 in the particulate fraction.

Further evidence for the ability of *Trichodesmium* to produce cobalamins comes from the widespread conservation of the *bluB* gene in *Trichodesmium* genomes sampled directly *in situ* and in the genome of *T. erythraeum* strain 2175 isolated 10 years after IMS101 (Figure 2). Upon searching a *Trichodesmium* metagenome sequenced from hand-picked natural colonies (Walworth et al., 2015), the *bluB* gene was detected via BLAST(Altschul et al., 1990) with 90% similarity and evalue ≤ 10^−123^. Accordingly, BLAST searches with this metagenomic BluB homolog against the NCBI non-redundant protein database returned IMS101 as its best hit followed by other BluB homologs in other bacterial phyla. These analyses demonstrate retention of the *bluB* gene in natural populations, perhaps suggesting that its maintenance has been selected for over time.

Taken together, these data suggest that *Trichodesmium* genus produces the DMB-producing enzyme BluB in culture and maintains the genetic capability *in situ*, thereby highlighting yet another possible critical keystone service provided by *Trichodesmium* to microbial communities. Further study investigating both cobalamin and pseudo-B_12_ cycling in *Trichodesmium* colonies is needed to better characterize this underappreciated source of cobalamin in the global oceans.

### Phylogenetics of btuR—B_12_ biosynthesis and salvage pathways

To date, biosynthesis of the corrin ring of the vitamin has been found to occur via either an oxygen-dependent (aerobic) or oxygen-independent (anaerobic) pathway (Rodionov, 2003). The production and use of (pseudo)cobalamin is predicted to predate oxygenic photosynthesis, and its genetic capacity is found in virtually all cyanobacterial genomes that have been analyzed (Heal et al., 2016). This suggests the pathway was likely present in the earliest members of the lineage and has been maintained through purifying selection over time. Based on phylogenetic analyses of several corrin ring biosynthesis genes of *T. erythraeum* strains IMS101 and 2175 (Figures S2–S4), *Trichodesmium* indeed retains the O_2_-independent pathway with all genes clustering deeply within a monophyletic cyanobacterial clade. This also holds true for genes involved in adenosylation following corrin biosynthesis to make adenosylcobalamin (Ado-CBL), an active form of cobalamin (Figures S5–S7).

Ado-CBL can either be salvaged via the adenosylation of exogenous corrinoids [e.g., cobinamide (Cbi)] or synthesized *de novo*. Both of these avenues require ATP:corrinoid adenosyltransferase encoded by the *btuR* gene (Rodionov, 2003). Transport of exogenous corrinoids is facilitated by the highly specific BtuBFCD system in many gram-negative bacteria (Escalante-Semerena, 2007) followed by further processing by the BtuR/CobA, CobU, CobS, and CobC enzymes (Figure 1; Rodionov, 2003; Escalante-Semerena, 2007). BtuR adenosylates a cobalamin precursor in the biosynthetic pathway, or other intermediates via the salvage pathway, to yield adenosylcobinamide (AdoCbi) (Rodionov, 2003; Fang et al., 2017; Figure 1). In the salvage pathway, catalysis by the bifunctional CobU enzyme (Tery_3432) yields Adenosyl-GDP-cobinamide (Figure 1), which is a true intermediate of the biosynthetic pathway (Woodson and Escalante-Semerena, 2003). Although *Trichodesmium* does not harbor the BtuBFCD transport system, *btuR, cobU, cobS*, and *cobC* homologs are present in the genome suggesting *Trichodesmium* may have the capacity to salvage exogenous cobalamin with transport potentially mediated by an alternative mechanism. Furthermore, transcription of all salvage genes was detected in addition to protein products of *cobC* and all three copies of *btuR* in *Trichodesmium* (Table S1), demonstrating salvage-specific enzyme activity in the presence of exogenous cobalamin.

*Trichodesmium* and other cyanobacteria contain three separate copies of genes retaining *btuR* domains, which are broadly distributed throughout the IMS101 genome. Two of these (Tery_0786, Tery_2088) are predicted to be 190 and 178 amino acids long, respectively, while the third (Tery_4685) harbors duplicated *btuR* catalytic domains and is roughly twice the size. The well-characterized *btuR/cobA* homologs of *Escherichia coli* and *Salmonella typhimurium* fall within the Tery_0786 cluster, suggesting this gene in *Trichodesmium* may also be involved in adenosylating cobinamide. While possessing two single-domain proteins is common among organisms beyond the Cyanobacteria, Tery_4685 and its homologs (all with duplicated domains) are almost exclusively unique to the phylum. The only two known exceptions to this include a chromatophore gene within the Eukaryote *Paulina chromatophora*, believed to have recently undergone an independent endosymbiotic event taking in a cyanobacterium (Nowack et al., 2008), and a cyanophage derived from *Prochlorococcus* that, of only 131 predicted proteins, possesses a dual-*btuR*-domain homolog (Sullivan et al., 2009). Aside from these extraordinary exceptions, this highly conserved, monophyletic distribution suggests that this intra-gene duplication event may have occurred after Cyanobacteria diverged, but very early in the phylum's lineage.

**Figure 4:**
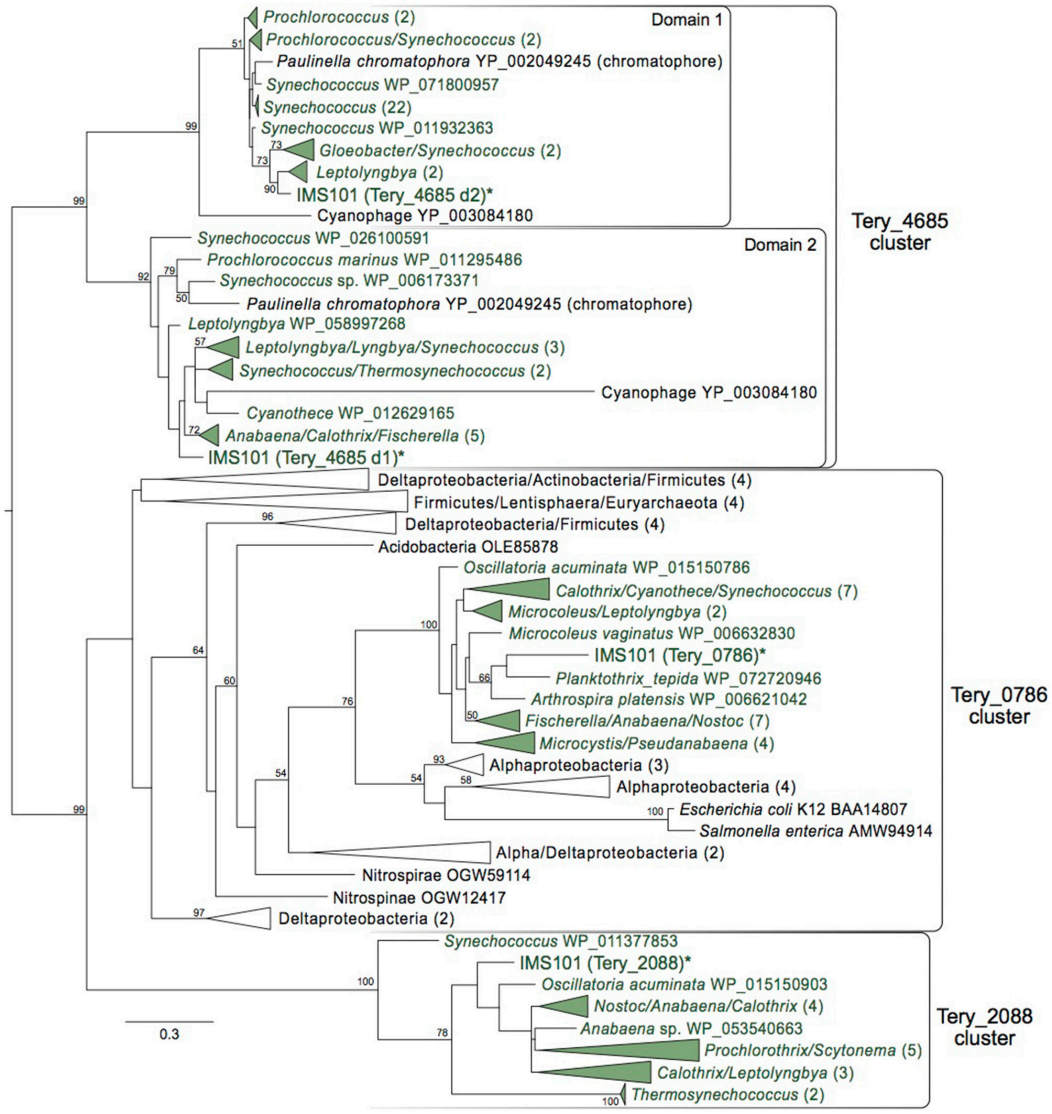
All three copies of *btuR* in IMS101 and maximum likelihood analysis of each catalytic domain of Tery_4685. Maximum likelihood phylogenetic tree (bootstraps = 100) of individual *btuR* domains (domain only). Cyanobacteria are colored green. Bootstrap values >50 are noted. Parentheses indicate how many taxa within a collapsed clade. Scale bar represents average substitutions per site.

The concurrent maintenance of all three of these phylogenetically distinct *btuR*-domain containing genes within the Cyanobacteria suggests they may carry out different roles—possibly with unique specificities for adenosylating varying compounds (e.g., cobinamide/cob(I)alamin/cob(I)yrinate diamide)—and stands as evidence of the successful duplication history this specific domain has experienced. To investigate this, each individual domain, as determined by its alignment to the encompassing protein family (pfam02572), was searched separately against the Refseq protein database (BLASTp) and phylogenetic analysis was performed. This revealed distinct, monophyletic clades for each of the co-occurring domains of Tery_4685 which, along with their conserved spatial relationship, provides further evidence they may have been under divergent yet connected evolutionary pressures (Figure 4).

Furthermore, we detected two distinct clades of homologs to Tery_2088 and Tery_0786 with the former being comprised entirely of Cyanobacteria, and the latter, while possessing a monophyletic clade of Cyanobacteria, being overall taxonomically diverse (Figure 4). While further investigation is required for any mechanistic insight into their roles, considering the conservation of Tery_2088 and Tery_4685 (sequence-wise and distribution-wise), they may be essential to the cyanobacterial eco-physiology.

### Association of B_12_ biosynthesis with iron bioavailability

De novo synthesis of vitamin B_12_ requires cobalt, whose total dissolved concentrations in the open ocean are in the picomolar range (5–105 pM L^−1^) (Knauer et al., 1982) These levels are even lower than those considering limiting for other bioactive trace elements like iron (~200 pM L^−1^) (Hutchins and Boyd, 2016). Consistent with those low levels of cobalt in the ocean, Panzeca et al. (2008) observed the enhancement of B_12_ production in cobalt amendment experiments, providing strong evidence that the synthesis of this vitamin could be limited by the availability of this trace element (Panzeca et al., 2008). However, the bacterial multifunctional enzyme CbiX^L^, which catalyzes the insertion of cobalt in B_12_ biosynthesis, contains an iron-sulfur cluster (4Fe-4S), thereby implicating B_12_ biosynthesis to be associated with iron availability (Leech et al., 2003). Archaea have been demonstrated to contain a comparatively smaller CbiX (CbiX^S^) enzyme ranging between 120 and 145 amino acids (aa), whereas the bacterial CbiX^L^ typically contains >300 aa's (Leech et al., 2003).

*Trichodesmium*, as well as other Cyanobacteria, contain two phylogenetically distinct copies (Figure S2) of the *cbiX* gene (Tery_4741 and Tery_4427)—of which the former contains an iron-sulfur cluster M*X*C*XX*C metal-binding motif (4Fe-4S) (Leech et al., 2003), while the latter does not (e.g., presumably not iron-sulfur containing; Figure S8). Both copies were indeed found to be transcribed indicating simultaneous expression, although their protein products were not detected (Table S1). Interestingly, only the longer Tery_4741 (347 aa) is homologous to annotated Archaea CbiX^S^ proteins harboring conserved M*X*C*XX*C motifs, while no homology was detected with the shorter Tery_4427 (293 aa) when searched against all Archaea via BLASTP. Both Tery_4741 and Tery_4427 contain CbiX and Sirohydrochlorin ferrochelatase (SirB) domains, yet Tery_4741 solely retains both the M*X*C*XX*C motif (Figure S8) and a C-terminal histidine-rich region typically diagnostic of CbiX^L^ (Brindley et al., 2003; Leech et al., 2003). SirB, involved in siroheme biosynthesis, shares homology with CbiX but lacks the C-terminal histidine-rich region resulting in a substantially shorter peptide (Leech et al., 2003). Interestingly, although Tery_4427 could have SirB functionality since it lacks the C-terminal histidine-rich region yet is homologous to Tery_4741 (Figure S8), homology was only detected between Tery_4741 and the *Bacillus megatarium* SirB homolog. Phylogenetic analysis places the Tery_4741 gene in a taxonomically mixed clade comprised of mostly Cyanobacteria and other gram-positive bacteria, while Tery_4427 resides within a clade comprised of nearly all Cyanobacteria except for several gram-positive ones (Figure S2). Additionally, many taxa contain multiple *cbiX* gene copies within a single genome. The presence of two phylogenetically distinct copies (Figure S2) may have implications for ecological strategy in microbial cobalamin production where reallocation of iron is enabled by functional substitution of the iron-sulfur-containing CbiX protein. Similar iron-sparing strategies such as replacement of the iron-sulfur protein electron carrier ferredoxin with non-iron-containing flavodoxin have been demonstrated in cyanobacteria and other photoautotrophs (Chappell and Webb, 2010; Chappell et al., 2012; Morrissey and Bowler, 2012). Since many different iron-containing proteins are involved in broad metabolic pathways throughout the cell such as photosynthesis, nitrogen fixation, and respiration, iron flux (e.g., allocation) to CbiX relative to other biochemical iron sinks remains to be determined. However, *Trichodesmium* does contain the B_12_-dependent ribonucleotide reductase (NrdJ; Tery_0428) essential for the reduction of ribonucleotides to deoxyribonucleotides in DNA synthesis implicating a critical role for cobalamin in core metabolism. Future studies examining both functional roles and enzyme efficiencies under co-limiting iron and cobalamin conditions are necessary to characterize these enzymes in the context of their ecophysiology.

Another line of evidence suggesting exogenous iron association to B_12_ biosynthesis is the fact that the *Trichodesmium* cobalt reductase orthologue to the *Salmonella* B_12_ flavodoxin (*fldA*; reciprocal best blast; evalue < 10^−42^), postulated to be responsible for the reduction of Cob(II)yrinic acid a,c-diamide to Cob(I)yrinic acid a,c-diamide (Figure 1; Fonseca and Escalante-Semerena, 2001), is the iron-stress flavodoxin gene *isiB* (Tery_1666)—which has been demonstrated to be directly regulated by iron bioavailability (Fonseca and Escalante-Semerena, 2001; Chappell and Webb, 2010; Walworth et al., 2016). It must be noted that *Trichodesmium* also contains another *fldA* that is not regulated by iron (Chappell and Webb, 2010), which could also possibly be involved in this reduction. Indeed, *Trichodesmium* is limited by iron across expansive ocean biomes (Sohm et al., 2011; Chappell et al., 2012; Hutchins and Boyd, 2016), which consequently limits new nitrogen inputs for primary production as a whole. Furthermore, since homologs of *isiB* are highly conserved in microbes possessing the anaerobic cobalamin biosynthesis pathway (Helliwell et al., 2016), these data suggest that B_12_ biosynthesis and thus overall environmental bioavailability could be influenced by limiting iron. Interestingly, the BioCyc database (https://biocyc.org) implicates another gene, Tery_4461, as the cobalt reductase while the KEGG database (http://www.genome.jp/kegg/pathway.html) implicates *isiB* (Tery_1666) as above. However, it is compelling that *isiB* is indeed the true ortholog to the empirically investigated *Salmonella* B_12_ *fldA* (see above), which opens the door for future research in distinguishing the roles of these enzymes relative to cobalamin metabolism. As previously noted (Gaudu and Weiss, 2000), mutations to *fldA* homologs in genetically tractable organisms (e.g., *E. coli*) have been lethal, thereby prohibiting a traditional genetic approach to determine whether *fldA* is redundant to co(II)rrinoid reduction for adenosylation or whether it is solely responsible. Nonetheless, these data and other previous studies (Gaudu and Weiss, 2000; Heal et al., 2016) implicate a connective role between two essential micronutrients via prokaryotic core metabolism, and suggest that iron availability could directly influence not only nitrogen fixation, but also B_12_ biosynthesis in *Trichodesmium*.

## Conclusions

We provide multiple lines of evidence both in culture and *in situ* that suggest *Trichodesmium* can transcribe and translate the full genomic pathway to biosynthesize and/or metabolize cobalamins. Our proof-of-concept meta-analyses from independent sources and analytical techniques open the door for more in-depth research into *Trichodesmium* B-vitamin dynamics. Since the synthesis of pseudo-B_12_ is an oxygen-independent pathway, *Trichodesmium* may have originally relied on pseudo-B_12_ biosynthesis in conjunction with early nitrogen fixation (another oxygen independent process). Hence, the *bluB* gene may have been acquired horizontally much later as evidenced by its clustering within Proteobacteria, although it is prohibitively difficult to confirm this from phylogeny alone. Future studies will be needed to specifically assess cobalamin vs pseudo-B_12_ production in *Trichodesmium*. Future experiments could include investigating isotopically labeled cobalamin- and iron- (co)-limiting molecular physiology with a range of biogeographically distinct isolates, in addition to analyzing B-vitamin dynamics directly from natural populations. Since large areas of the ocean are depleted in both B_12_ and bioavailable nitrogen, these data and other future experiments may highlight an additional keystone role that *Trichodesmium* populations could serve in oceanic biomes. Importantly, this role would distinguish *Trichodesmium* from other sympatric cyanobacteria that solely produce pseudo-B_12_, including other nitrogen fixers as mentioned above. Hence, cobalamin production may have aided in the ecological success of *Trichodesmium* if cobalamins are indeed used as a “currency” in exchange for other growth factors supplied by its associated epibionts, as potentially suggested by B_12_ auxotrophy in IMS101-associated epibiotic heterotrophs (Lee et al., 2017a; Romine et al., 2017). Further research with *Trichodesmium* isolates devoid of cyanobacterial epibionts is needed to investigate whether *Trichodesmium* can simultaneously produce both cobalamin and pseudo-B_12_, thereby potentially giving them the flexibility to exploit different B_12_ ecological niches in the oceans. Moreover, *Trichodesmium* and other cyanobacteria possess three copies of the *btuR* gene, predicted to be involved in *de novo* synthesis and corrinoid scavenging. Two of these three copies appear to be unique to cyanobacteria. Further characterization of these gene copies may provide critical insight into cyanobacterial B_12_ ecophysiology relative to other B_12_-producing microbes. Finally, *cbiX* (Tery_4741) and *isiB* (Tery_1666) genes within the cobalamin biosynthetic pathway may both be subject to influence by iron, thereby highlighting a potentially unrecognized role for iron limitation in simultaneously impacting *Trichodesmium* photosynthesis, nitrogen fixation, and B_12_ biosynthesis. Experiments examining *Trichodesmium* iron limitation in the context of cobalamin limitation could reveal allocation dynamics of intracellular iron among critical iron-requiring metabolic pathways, and may indicate if the availability of this trace metal could potentially limit B_12_ synthesis *in situ*. Any preferential iron allocation strategies that may be employed by *Trichodesmium* under these conditions are currently unknown, but are certainly critical to understanding B_12_ oceanic cycling if this diazotrophic cyanobacterium is indeed also a major euphotic zone source for cobalamins in the global oceans.

## Availability of data and materials

Raw reads have been deposited in NCBI's Gene Expression Omnibus (Edgar et al., 2002) and are accessible through GEO Series accession number GSE94951 (https://www.ncbi.nlm.nih.gov/geo/query/acc.cgi?acc=GSE94951). The raw read files used in this study have accession numbers GSM2492342 and GSM2492343. Experimental data are also available through the Biological and Chemical Oceanography Data Management Office (www.bco-dmo.org/project/551230 and www.bco-dmo.org/project/724451).

Protein spectral counts were downloaded directly from a previously published proteome study using these same IMS101 cell lines (Walworth et al., 2016).

## Subject categories

Integrated genomics and post-genomics approaches in microbial ecology.

## Author contributions

DH, NW, ML, CS, PQ, F-XF, MS, EW, and SS-W: helped design and carry out the research; NW, DH, ML, MS, EW, and SS-W: were involved in writing and editing the paper.

### Conflict of interest statement

The authors declare that the research was conducted in the absence of any commercial or financial relationships that could be construed as a potential conflict of interest.
