# Supplementary material for: Functional Genomics and Phylogenetic Evidence Suggest Genus-Wide Cobalamin Production by the Globally Distributed Marine Nitrogen Fixer Trichodesmium
Source: Front Microbiol. 2018 Feb 13;9:189. doi: 10.3389/fmicb.2018.00189 (PMC5816740; doi:10.3389/fmicb.2018.00189)
Supplement: Supplementary file 1 [file DataSheet1.pdf]

Supplementary Information

Particulate B-vitamin analyses

Briefly, the cells on the filters were lysed by bead beating in an acidic methanol lysis solution followed by a chloroform liquid phase extraction to reduce signal suppression and negative matrix effects. Analysis of samples was conducted using a Thermo Scientific Quantum Access electrospray ionization triple quadrupole mass spectrometer, coupled to a Thermo Scientific Accela High Speed Liquid Chromatography (LC/MS) system. The LC system used a stable-bond C_{18} reversed-phase column (Discovery HS C_{18} 10cm × 2.1mm, 5µm column, Supelco Analytical), with a methanol:water gradient program (Sanudo-Wilhelmy et al., 2012). Triple injections of each sample were used to increase precision. Quantification was conducted using a stable isotopically labeled riboflavin as an internal standard. Total cobalamin was calculated by summing the mean observed concentrations of adenosyl-, methyl-, hydroxy-, and cyano-cobalamin. The reported standard deviation is the square root of the sum of the variances of each form of cobalamin (Fig. S1). The units used are picomolar (e.g., picomoles of particulate cobalamin per liter of culture).

Consortia RNA read processing and annotation

Reads not recruited to IMS101's reference genome were scanned with SortMeRNA v.2.0 with default settings in order to identify and remove any remaining ribosomal RNA reads (Kopylova et al., 2012). Samples were then concatenated together and co-assembled with Trinity v.2.4.0 with default settings (which included normalization in this version) (Haas et al., 2013). Coding sequences were then identified in these assembled transcripts via Prodigal v.2.6.2 with the –p flag set to 'meta' (Hyatt et al., 2012). Duplicate coding sequences and contaminants were removed with dedupe.sh, available within the BBMap suite of tools (Bushnell, B. sourceforge.net/projects/bbmap/). These remaining coding sequences were utilized as our

reference library for recruiting the original metatranscriptomic reads from each individual sample using Bowtie2, and read counts were then converted to transcripts per million by first normalizing to coding sequence length and then by sample library size. Amino acid sequences of coding sequences were annotated with Kyoto Encyclopedia of Genes and Genomes (KEGG) orthologs (KOs) (Kanehisa et al., 2016). B_{12}-related genes discussed herein were searched against NCBI's RefSeq protein database via BLASTp to identify taxonomy (evalue < 10^{-20}).

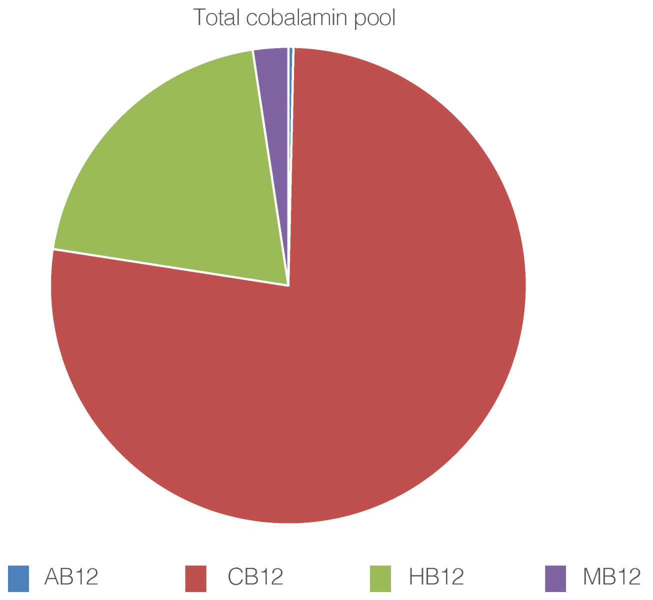

Fig. S1 Distribution of different forms of cobalamin making up the total pool from cultures of *Trichodesmium* IMS101. Cobalamin was extracted from filtered culture samples, and analyzed using LC/MS. The total cobalamin pool is represented including adenosyl- (AB), methyl- (MB), hydroxy- (HB), and cyano- (CB) cobalamin pools and had a mean of 58.4 (+/- 27.1) pM (e.g., picomoles of particulate cobalamin per liter of culture).

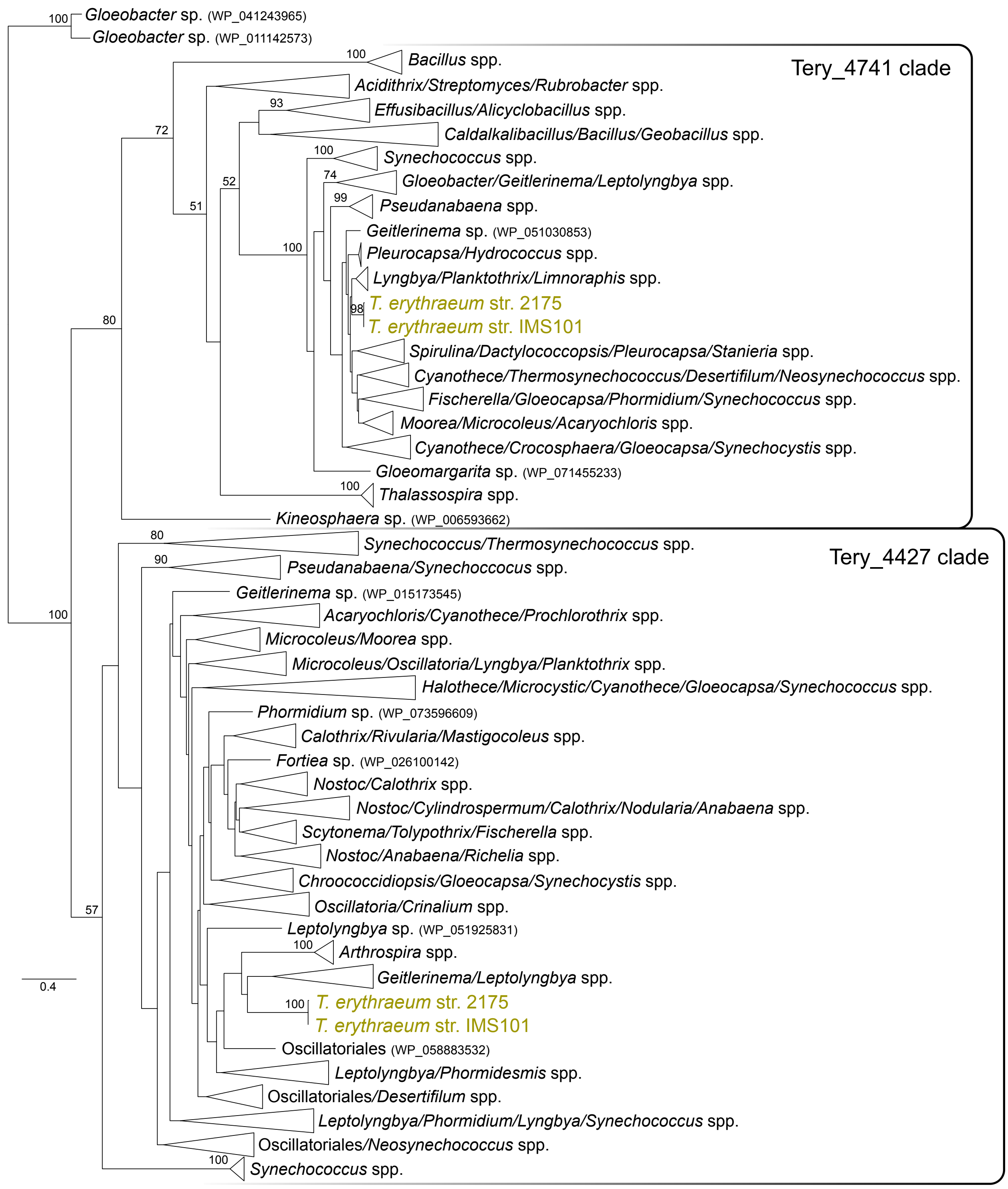

Fig. S2 Maximum likelihood phylogenetic analysis of IMS101 and 2175 *cbiX* genes (Tery_4741 and Tery_4427) and their closest homologs with 100 bootstraps.

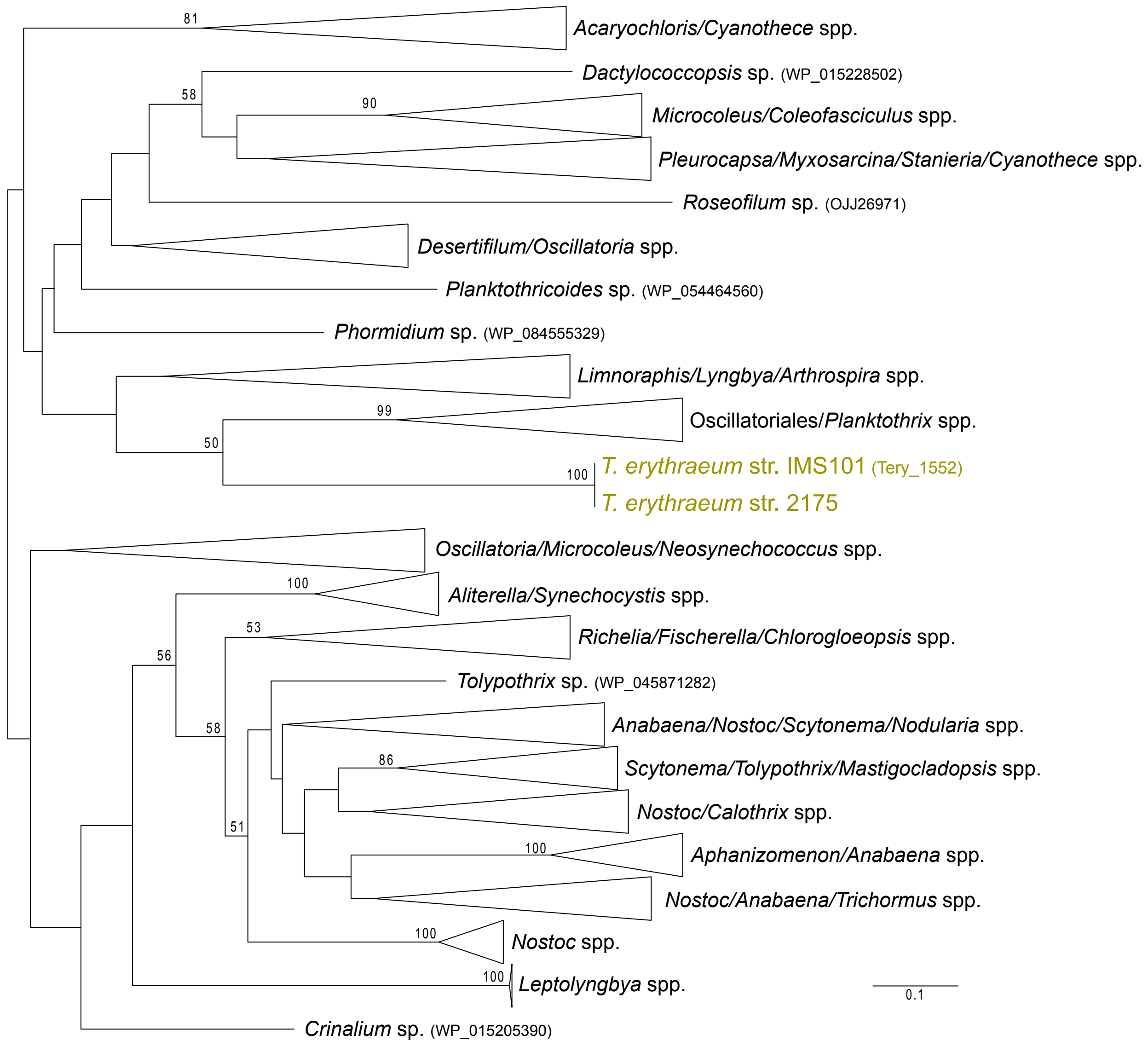

Fig. S3 Maximum likelihood phylogenetic analysis of the *Trichodesmium cbiF* and its closest homologs with 100 bootstraps.

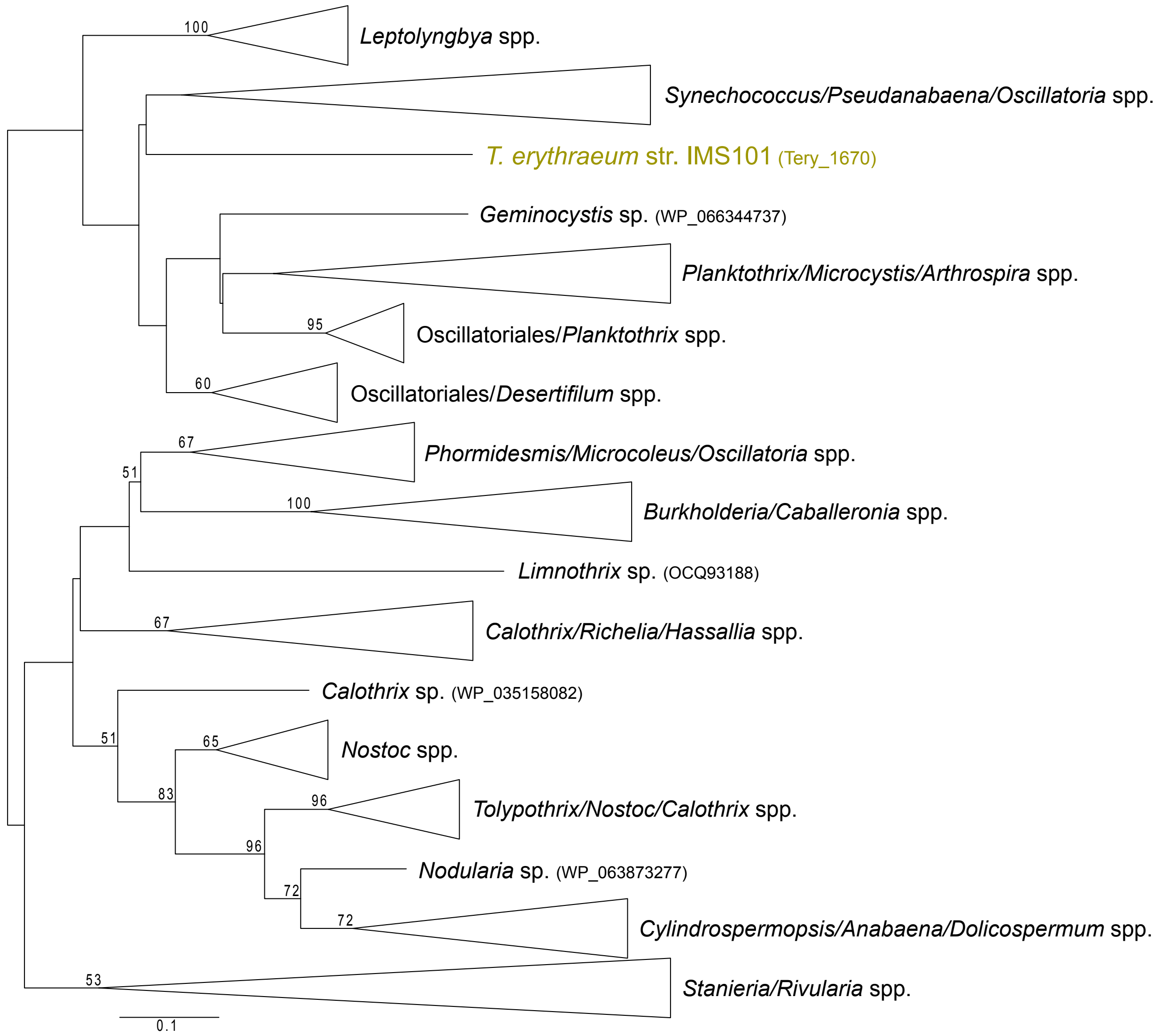

Fig. S4 Maximum likelihood phylogenetic analysis of the *Trichodesmium cbiC* and its closest homologs with 100 bootstraps.

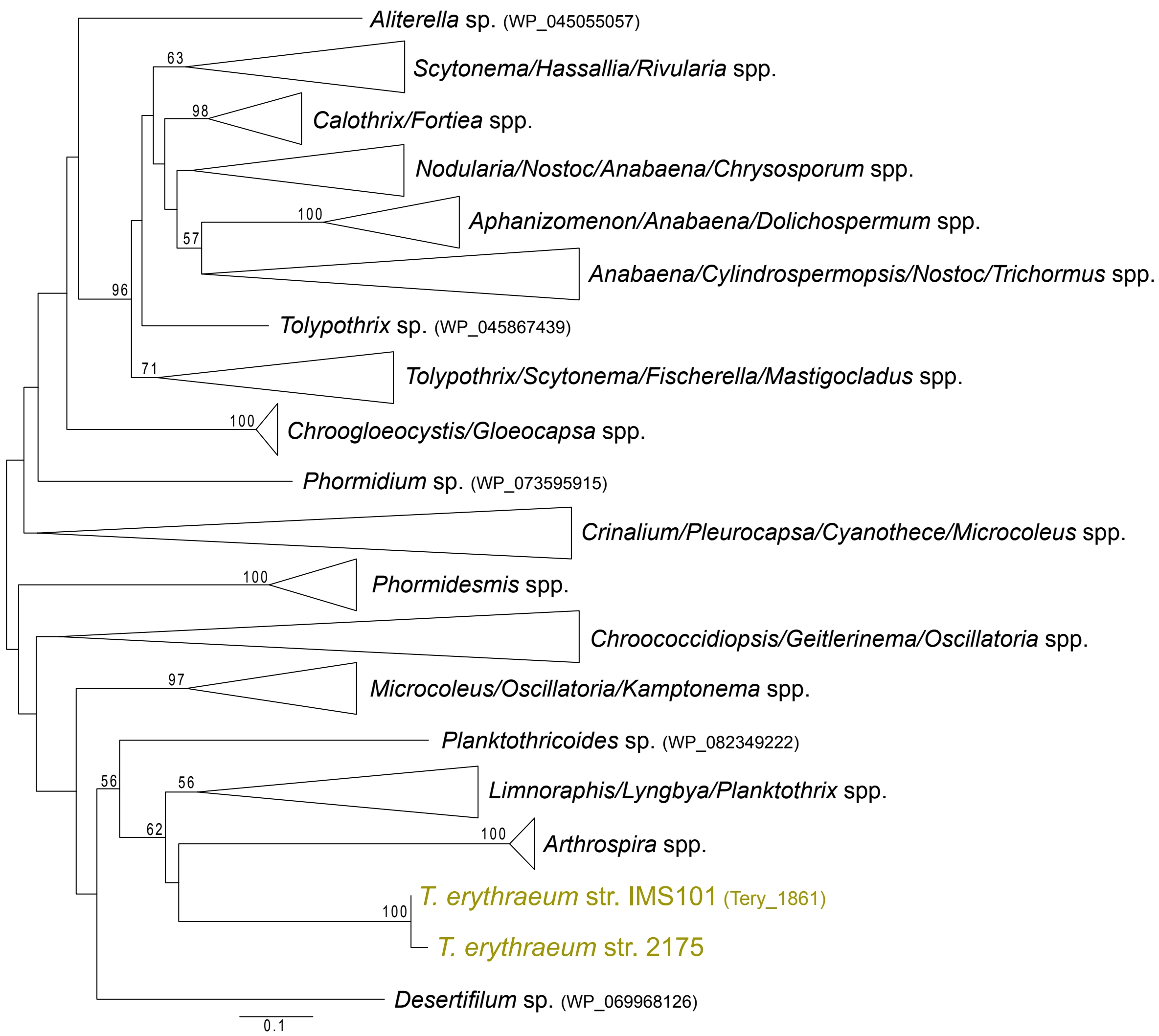

Fig. S5 Maximum likelihood phylogenetic analysis of the *Trichodesmium cobD* and its closest homologs with 100 bootstraps.

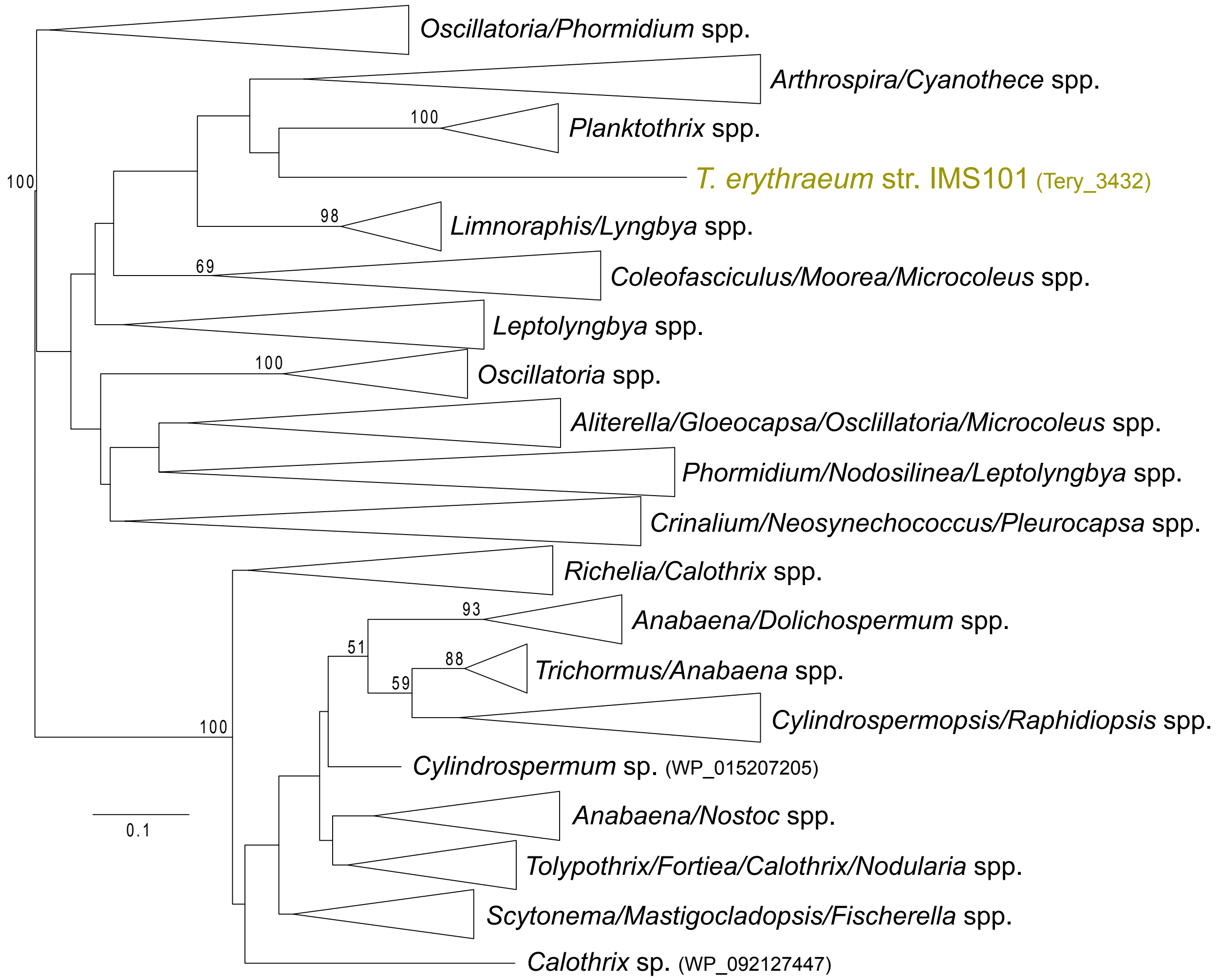

Fig. S6 Maximum likelihood phylogenetic analysis of the *Trichodesmium cobP* and its closest homologs with 100 bootstraps.

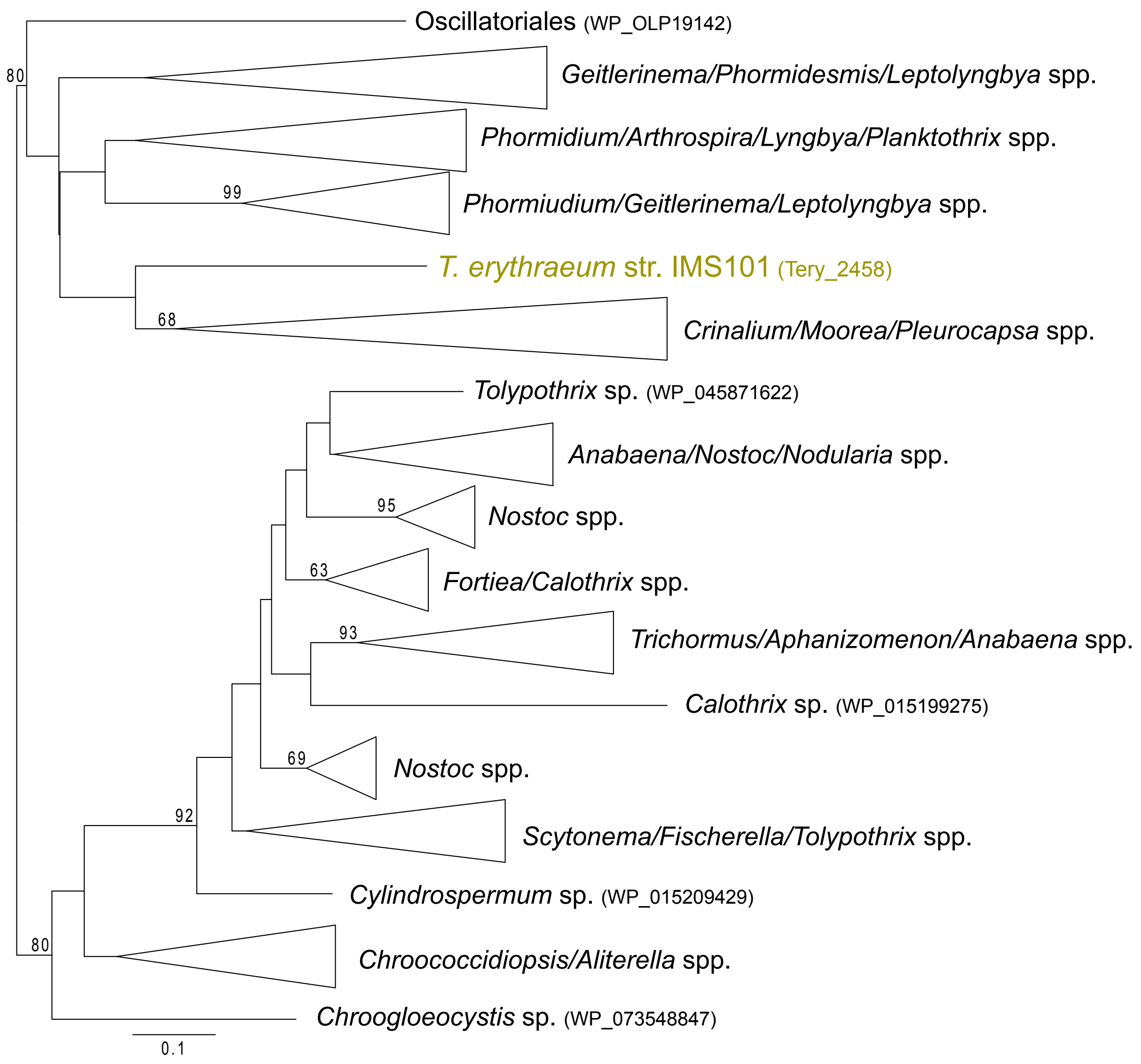

Fig. S7 Maximum likelihood phylogenetic analysis of the *Trichodesmium cobS* and its closest homologs with 100 bootstraps.

```
Tery_4741       MQYSNQNPKIKTQKILTSTIEKSHLYTEEKLESLPLQRPLLLIGHGTRDSQGK-------
Tery_4427       MNFVND------------------LFMNNYLST------YFLVSHGSRDPRPKLELQTLA
                *:: *:                  *: :: *.:       :*:.**:**.. *

Tery_4741       ------ETFLDFVAAYQNLDKSRPVIPCFLELTKPTIQEGVDKCVEQ----GYTELSALP
Tery_4427       NLLSKQTTLSKYFRQHNNILRYPMITTGVLELGPKPLHEQIEDFWQSIRALHISQIQIIP
                       *: .:.  ::*: .   : . .***   .::* ::.  :.      :::. :*

Tery_4741       ILLFAARHNKFDITNELDRARQR--------------------------HPQVKFYYGRH
Tery_4427       LFLLPGVHVTEDIPAEIQIFRERVKANATSKIVLHEEKSHLKLEKKLEINIPIKVNLYPY
                ::*:.. * . **. *::  *:*                          :  :*.    :

Tery_4741       FGITPKIIELWRSRLKELDKTNFNSSETVLLFVGRGSSDPDANGDVYKLARMLWEG----
Tery_4427       IGSHPKMVNLLATKMTSVIAEAW-------VIISHGSRRTESNEVVEKISQFLSSSCKVL
                :*  **:::*  :.:..:    :       :::..**  .::*  * *::.:* ..

Tery_4741       --SGYLTVETCFIGITHPRLEEGFRRARLYQPKRIMVLPYFLFTGVLVKKIFDITAQQQE
Tery_4427       VCTAYWSVPPDLKSRVDILMKQGY--------KKIGILPYFLFNGGITDAIADTVNQLSQ
                  :.* :* . : . .   :::*:        *.* :******.* :.. * * . * .:

Tery_4741       QYPDISMTCLPEIGAHPTLLELLREREIETQLGEVKMNCEMCKFRLVTVTNREHSHGHSH
Tery_4427       IYPTIQFHMTTPLGPTEELACLVAD--------------------LVS-----SSQG---
                 ** *.:   . :*.   *  *: :                    **:      *:*

Tery_4741       HYHDHSHSLNIPEHSHSVVDPYAEPEKYHQRIWQVP
Tery_4427       ------------------------------------
```

Fig. S8 Multiple sequence alignment of the two CbiX copies from Fig. S2. The metal-binding M*X*C*XX*C motif is denoted in grey in Tery_4741 and is absent in Tery_4427.

Supplementary Data

Table S1: These are the TMM-normalized (Robinson and Oshlack, 2010) expression levels of B_{12} biosynthesis/salvage genes that were sampled midday from the *Trichodesmium* long-term experiment (Walworth et al., 2017). R1 and R2 stand for biological replicate 1 and 2 of the r380 treatment in Walworth et al. 2017 in which cells were grown in nutrient-replete, Aquil medium in 380 ppm CO_2.

		**R1**	**R2**
**Tery_4461**	bluB	11.7559833	9.87497621
**Tery_0786**	btuR	43.16650118	36.25242944
**Tery_0517**	cbiA	15.82334359	19.06511067
**Tery_1861**	cbiB	98.79749358	113.6174556
**Tery_1670**	cbiC	8.633300236	7.312342563
**Tery_0905**	cbiD	19.05099079	14.6467768
**Tery_0210**	cbiE	45.23954288	39.65454653
**Tery_1552**	cbiF	43.6388398	43.29967197
**Tery_4366**	cbiG	51.90476555	58.36619049
**Tery_1175**	cbiH	20.41552457	24.89731138
**Tery_0768**	cbiL	18.00134943	19.06511067
**Tery_2739**	cbiJ	39.33531019	48.15983924
**Tery_3957**	cbiP	57.73027513	67.71096663
**Tery_1288**	cbiT	36.55376057	34.59555424
**Tery_4741**	cbiX	99.24359116	113.7058223
**Tery_4427**	cbiX	17.26660047	18.4686356
**Tery_2181**	cobC	507.5803236	478.8369337
**Tery_2458**	cobS	14.66873809	15.61881025
**Tery_3432**	cobU	27.36939862	28.82962853
**Tery_3325**	cysG	29.20627101	27.96805343
**Tery_1666**	isiB	1098.948269	1002.69669
**Tery_2088**	btuR	571.2481888	585.3839262
**Tery_4685**	btuR	155.9785573	126.5139721

Table S2: These are the normalized spectral counts of B_{12} biosynthesis/salvage proteins that were sampled midday from the *Trichodesmium* long-term experiment (Walworth et al., 2016). R1, R2, and R3 stand for biological replicate 1, 2, and 3 of the r380 treatment in Walworth et al. 2016 in which cells were grown in nutrient-replete, Aquil medium in 380 ppm CO_2.

**Id**		**R1**	**R2**	**R3**
**Tery_3325**	CysG	1.8056	2.777	1.7974
**Tery_2181**	CobC	32.501	31.473	35.947
**Tery_3957**	CbiP	0.9028	0.92568	0.89868
**Tery_4461**	BluB	3.6112	3.7027	3.5947
**Tery_1666**	IsiB	65.905	63.872	65.604
**Tery_0786**	BtuR	3.6112	1.8514	3.5947
**Tery_4366**	CbiG	0.9028	1.8514	0.89868
**Tery_1552**	CbiF	2.7084	3.7027	2.696
**Tery_1288**	CbiT	0.9028	1.8514	2.696
**Tery_2088**	BtuR	6.3196	6.4797	5.3921
**Tery_4685**	BtuR	22.57	21.291	21.568

Table S3: These are the TMM-normalized (Robinson and Oshlack, 2010) expression levels of the B_{12}-dependent *metH* and *nrdJ* and B_{12}-independent *metE* that were sampled midday from the *Trichodesmium* long-term experiment (Walworth et al., 2017). R1 and R2 stand for biological replicate 1 and 2 of the r380 treatment in Walworth et al. 2017 in which cells were grown in nutrient-replete, Aquil medium in 380 ppm CO_2.

RNA		**R1**	**R2**
**Tery_0428**	nrdJ	164.8174297	149.0907626
**Tery_1073**	metH	430.0622843	413.826827
**Tery_0847**	metE	3971.197808	4105.551806

Table S4: These are the normalized spectral counts of the B_{12}-dependent *metH* and *nrdJ* and B_{12}-independent *metE* that were sampled midday from the *Trichodesmium* long-term experiment (Walworth et al., 2016). R1, R2, and R3 stand for biological replicate 1, 2, and 3 of the r380 treatment in Walworth et al. 2016 in which cells were grown in nutrient-replete, Aquil medium in 380 ppm CO_2.

Protein		**R1**	**R3**	**R3**
**Tery_1073**	MetH	24.376	24.993	35.049
**Tery_0847**	MetE	146.25	161.07	145.59

Table S5: These are in transcripts per million for the community only (w/o *Trichodesmium* transcripts). These data are from the *Trichodesmium* consortia metatranscriptome as described in Lee et al. 2017(Lee et al., 2017).

	R1	R2	Gene symbol	KO_ID	Kegg deffinition	Blast tax
**CDS_44539**	24.94	22.41	MMAB/pduO/ btuR	K00798	cob(I)alamin adenosyltransferase	Bacteroidetes (Phaeodactyliba cter xiamenensis)
**CDS_55643**	0.09	0.11	MMAB/pduO/ btuR	K00798	cob(I)alamin adenosyltransferase	Bacteroidetes (Lewinella cohaerens)
**CDS_50601**	0	0.83	MMAB/pduO/ btuR	K00798	cob(I)alamin adenosyltransferase	Alphaproteobact eria (Ruegeria atlantica)
**CDS_71531**	0	0.66	MMAB/pduO/ btuR	K00798	cob(I)alamin adenosyltransferase	Synechococcus
**CDS_17881**	0.13	2.09	btuR/cobA	K19221	cob(I)alamin adenosyltransferase	Synechococcus
**CDS_28823**	0.19	0	btuR/cobA	K19221	cob(I)alamin adenosyltransferase	Synechococcus
**CDS_35672**	0	1.03	btuR/cobA	K19221	cob(I)alamin adenosyltransferase	Synechococcus
**CDS_62206**	1.05	0.16	btuR/cobA	K19221	cob(I)alamin adenosyltransferase	Synechococcus
**CDS_51740**	1.15	3.86	cbiB/cobD	K02227	adenosylcobinamide-phosphate synthase	Synechococcus

References

Haas, B. J., Papanicolaou, A., Yassour, M., Grabherr, M., Blood, P. D., Bowden, J., et al. (2013). De novo transcript sequence reconstruction from RNA-seq using the Trinity platform for reference generation and analysis. *Nature Protocols* 8, 1494–1512. doi:10.1038/nprot.2013.084.

Hyatt, D., LoCascio, P. F., Hauser, L. J., and Uberbacher, E. C. (2012). Gene and translation initiation site prediction in metagenomic sequences. *Bioinformatics* 28, 2223–2230. doi:10.1093/bioinformatics/bts429.

Kanehisa, M., Sato, Y., Kawashima, M., Furumichi, M., and Tanabe, M. (2016). KEGG as a reference resource for gene and protein annotation. *Nucleic Acids Res* 44, D457–D462. doi:10.1093/nar/gkv1070.

Kopylova, E., Noé, L., and Touzet, H. (2012). SortMeRNA: fast and accurate filtering of ribosomal RNAs in metatranscriptomic data. *Bioinformatics* 28, 3211–3217. doi:10.1093/bioinformatics/bts611.

Lee, M. D., Walworth, N. G., McParland, E. L., Fu, F.-X., Mincer, T. J., Levine, N. M., et al. (2017). The *Trichodesmium* consortium: conserved heterotrophic co-occurrence and genomic signatures of potential interactions. *ISME J.* doi:10.1038/ismej.2017.49.

Robinson, M. D., and Oshlack, A. (2010). A scaling normalization method for differential expression analysis of RNA-seq data. *Genome Biol.* 11, R25. doi:10.1186/gb-2010-11-3-r25.

Sanudo-Wilhelmy, S., Cutter, L. S., Durazo, R., Smail, E. A., Gomez-Consarnau, L., Webb, E. A., et al. (2012). Multiple B-vitamin depletion in large areas of the coastal ocean. *Proc Natl Acad Sci USA* 109, 14041–14045. doi:10.1073/pnas.1208755109/-/DCSupplemental.

Walworth, N. G., Fu, F.-X., Lee, M. D., Cai, X., Saito, M. A., Webb, E. A., et al. (2017). Nutrient co-limited *Trichodesmium* as nitrogen source or sink in a future ocean. *Appl. Environ. Microbiol.*, AEM.02137–17–16. doi:10.1128/AEM.02137-17.

Walworth, N. G., Fu, F.-X., Webb, E. A., Saito, M. A., Moran, D., Mcllvin, M. R., et al. (2016). Mechanisms of increased *Trichodesmium* fitness under iron and phosphorus co-limitation in the present and future ocean. *Nat. Commun.* 7, 12081. doi:10.1038/ncomms12081.
